# Evaluation of the enzymatic digestibility of paper industry byproducts

**DOI:** 10.1186/1753-6561-5-S7-P117

**Published:** 2011-09-13

**Authors:** Patrícia Silva, Washington Luiz Magalhães, Cristiane Helm, Edson Lima, Dayanne Mendes, Tielidy Lima

**Affiliations:** 1 Embrapa Forestry, Brazil; 2 Federal University of Paraná – UFPR, Brazil

## Background

Researches concerning renewable energy sources and wastes recycling are strategic to ensure energetic and environmental sustainability of the planet. In this scenario, biofuels (including bioethanol) have attracted increasing attention.

Most ethanol is currently produced from sugar- and starch-based feedstocks, such as sugar cane in Brazil and corn in the U.S.A. However, lignocellulosic biomass also presents great potential for being used as an alternative raw material. Unlike ordinary feedstocks, sugars from cellulosic biomass are not readily available for fermentation and conversion to ethanol. A preliminary pretreatment step is necessary in order to remove the lignin that involves the structures of cellulose and hemicellulose, acting as a barrier. In the following step, fermentable sugars can be released by enzymatic hydrolysis of polysaccharides. Finally, monosaccharides can be converted into ethanol by anaerobic fermentation [1,2].

This process allows the use of forest wastes to produce ethanol. Besides adding value to these byproducts, this action could contribute to reducing environmental problems related to waste disposal.

Several solid byproducts from pulp and paper industry have a high conversion potential into ethanol [3-7], since they have high carbohydrate content and susceptibility to hydrolysis process. This is due to the fact they have already undergone a previous treatment of the fibers. One example is the sludge generated in wastewater treatment of paper recycling industries, which is composed primarily of cellulose and ash. Although studies have been conducted focusing recycling and reusing, currently its primary destination has been the disposal in landfills, causing high costs and requiring large storage areas. As this product usually has high content of moisture, the production of wet sludge can reach one ton per ton of paper produced [8]. Therefore, suitable utilization of such waste would result in significant economic benefits to the pulp and paper sector.

The aim of this study was to evaluate the enzymatic digestibility of byproducts generated by paper industry, including samples of recycled paper sludge and bleached and unbleached pulps.

## Methods

The “recycled paper sludge” was a pressed solid material resulting from the wastewater treatment process of a paper recycling mill. Two samples obtained from Kraft pulping process were also used: an unbleached pulp and a bleached one. All materials were ground in a blender before use. Water and ash contents were determined gravimetrically, according to international standards.

Samples were suspended in 0.05 M sodium citrate buffer, pH 4.8, to obtain an initial solids concentration of about 5 g.L^-1^ (dry basis). A mixture of two commercial enzyme preparations was added: cellulase 2.25 mL.L^-1^ (Celluclast 1.5 L), and cellobiase 1.13 mL.L^-1^ (Novozyme 188). Samples were incubated at 50 °C in an orbital shaker at 250 rpm for 24 h. This hydrolysis time was selected based on a recent study that has demonstrated little effect of time on the process yield over 24 hours [9]. Tests were performed in triplicate for each sample and the concentrations of reducing sugars were estimated by the dinitrosalycilic acid method.

## Results and conclusions

Recycled paper sludge presented high contents of moisture (66.2 %) and ash (20.6 %, wet basis). Inorganic fraction probably includes additives such as kaolin, calcium carbonate and titanium dioxide, as well as metals from printing inks. Moisture and ash fractions were around 7.0 % and 0.3 % for bleached pulp and 22.5 % and 1.1 % for unbleached pulp.

Cellulose conversions estimated from the hydrolysis trials were: 82.1 % for the bleached pulp, 69.5 % for the sludge and 27.2 % for the unbleached pulp. As expected, the sample that was not subjected to the bleaching process showed lower susceptibility to hydrolysis, probably due to its higher residual lignin content that hinders the enzyme action. The other materials showed relatively high saccharification efficiencies because they have already been “pretreated” during the production process itself. Although paper sludge have presented lower conversion than the bleached pulp, the presence of inorganic compounds seemed to cause no significant inhibition of the saccharification process.

Therefore, the present study suggests the technical feasibility of using the materials tested as a source of monosaccharides for ethanol production, without requiring an additional step of pretreatment. Special interest is focused in the use of sludge from the paper recycling process, adding value to this byproduct and minimizing environmental impacts.

## References

[B1] Hamelinck C Hooijdonk G Faaij A Ethanol from lignocellulosic biomass: techno-economic performance in short-, middle- and long-term Biomass and Bioenergy 2005 28 384 410 10.1016/j.biombioe.2004.09.002

[B2] Taherzadeh MJ Karimi K Enzyme-based hydrolysis processes for ethanol from lignocellulosic materials: a review BioResources 2007 2 707 738

[B3] Lark N Xia Y Qin C Gong C Production of ethanol from recycled paper sludge using cellulase and yeast, *Kluveromyces marxianus* Biomass and Bioenergy 1997 12 135 143 10.1016/S0961-9534(96)00069-4

[B4] Ballesteros M Oliva JM Manzanares P Negro MJ Ballesteros I Ethanol production from paper material using a simultaneous saccharification and fermentation system in a fed-batch basis World J Microbiol Biotechnol 2002 18 559 561 10.1023/A:1016378326762

[B5] Fan Z South C Lyford K Munsie J Walsum Van P Lynd LR Conversion of paper sludge to ethanol in a semicontinuous solids-fed reactor Bioprocess Biosyst Eng 2003 26 93 101 10.1007/s00449-003-0337-x 14615931

[B6] Marques S Alves L Roseiro J Girio F Conversion of recycled paper sludge to ethanol by SHF and SSF using *Pichia stipitis* Biomass and Bioenergy 2008 32 400 406 10.1016/j.biombioe.2007.10.011

[B7] Zhang J Lynd LR Ethanol production from paper sludge by simultaneous saccharification and co-fermentation using recombinant xylose-fermenting microorganisms Biotechnol Bioeng 2010 107 235 44 10.1002/bit.22811 20506488

[B8] Foelkel C Resíduos Sólidos Industriais do Processo de Fabricação de Celulose e Papel de Eucalipto. Parte 03: Lodos & Lodos Eucalyptus OnLine Book; 2010 1 191 http://www.eucalyptus.com.br/eucaliptos/PT20_LODOS.pdf

[B9] Helm CV Magalhães WLE Lima EA Silva PR Hoffmann K Higa A Mendes D Time influence in the enzymatic saccharification of cellulose pulp samples (submitted paper) UFRO Tree Biotechnology 2011

